# Serum α-KL, a potential early marker of diabetes complications in youth with T1D, is regulated by miRNA 192

**DOI:** 10.3389/fendo.2022.937093

**Published:** 2022-08-05

**Authors:** Zhenwei Gong, Pedro A. Pagán Banchs, Ye Liu, Haoyi Fu, Vincent C. Arena, Erick Forno, Ingrid Libman, Jacqueline Ho, Radhika Muzumdar

**Affiliations:** ^1^ University of Pittsburgh Medical Center, Children’s Hospital of Pittsburgh, PA, United States; ^2^ Department of Pediatrics, University of Pittsburgh, PA, United States; ^3^ Department of Biostatistics, School of Public Health, University of Pittsburgh, PA, United States

**Keywords:** type 1 diabetes mellitus, Klotho, miR-192, diabetic nephropathy, biomarker

## Abstract

Despite the wealth of information on biomarkers of diabetes complications in adults with type 1 diabetes, data in the pediatric population is limited. Diabetic nephropathy (DN), the leading cause of mortality in type 1 diabetes T1D), could be potentially missed in youth, as albuminuria, the current “gold” standard, may be transient and may not reflect permanent renal impairment. Soluble alpha KL has emerged as a potential marker of early diabetic nephropathy. Seventy-nine pediatric patients with type 1 diabetes meeting ISPAD criteria for nephropathy screening were consecutively recruited (90% Caucasian, 51% male, mean age 16.1 ± 3.1 years, duration of T1D 7.2 ± 3.9 years, 2-year average HbA1c 8.0 ± 1.3%, and serum and urine samples were collected for analysis. Serum Klotho (KL) and circulating miRNA levels of select miRNA involved in the pathogenesis of DN were estimated. KL had a strong inverse correlation with diabetes duration and HbA1c, two important risk factors in the development of diabetes complications. Serum miR-192 were negatively associated with KL among children with prolonged duration of diabetes (≥12 years) after adjustment for age and sex. In cell culture, overexpression of miR-192 significantly downregulated KL mRNA and protein levels, and reduced KL levels in the media. miR-192 mimic reduced luciferase activity in a reporter containing the KL 3’ UTR (60% compared to controls, p<0.01), and the inhibitor rescued it. Deletion of a potential binding site for miR-192 in the KL 3’UTR completely abolished the effect of miR-192 in the reporter assay, suggesting that KL is a direct target gene of miR-192. Overexpression of miR-192 significantly increased oxidative stress (MDA) and expression of inflammatory and senescence markers IL-6 and p16. Inhibition of miR-192 significantly reduced levels of MDA, IL-6 and p16. In summary, we demonstrate an increase in miR-192 and a decrease in KL levels in children with prolonged duration of T1D. We demonstrate a novel role for miR-192 in directly regulating KL levels, and through that, senescence and oxidative stress, key pathological processes in the development of DN. miR-192 and/or KL levels are altered with severity and duration of diabetes and could serve as early biomarkers for DN.

## Introduction

Diabetic nephropathy (DN) is a significant complication of type 1 and type 2 diabetes. DN is characterized by persistent albuminuria and a progressive decline in renal function. It is reported that the prevalence of DN is between 20% to 50% among those living with diabetes (1), and clinical studies have shown that DN is the most frequent cause of end-stage kidney disease (ESKD) in most countries (1). In the U.S., DN accounted for nearly 50% of all patients (more than 58,000 people) with ESKD (United States Renal Data System, 2018, NIH, NIDDK). The annual prevalence of DN in the pediatric population has also been increasing significantly, with one reported study citing an increase from 1.16% to 3.44% from 2002-2013 (2).

Alpha-Klotho (KL), an obligate co-receptor molecule for fibroblast growth factor (FGF) 23 function, was originally identified as an anti-aging protein (3). KL was found to be expressed in multiple tissues, with highest expression in the kidneys (4–6). Mice lacking KL display multiple aging-like phenotypes including vascular calcification and osteoporosis and died prematurely at around two to three months of age (4), and overexpression of the KL gene extends life span in mice (7). Clinical and basic research have provided evidence that KL is involved in many human diseases including cardiovascular disease, osteoporosis, cancers, and acute and chronic kidney diseases (8). The effects of KL are mediated through regulation of biological processes and signaling pathways including oxidative stress, inflammation, and fibrosis (3). Renal expression of KL as well as circulating KL levels are severely decreased in patients with chronic kidney disease (9) and in experimental animal models of kidney disease (10–13) including in diabetic db/db mice. Most of the published evidence is from adults with diabetes and kidney disease, while studies examining circulating KL levels in children with type 1 diabetes (T1D) are very limited. In a cross-sectional single center study from Poland, sKL was lower in children with T1D than in the controls and correlated with HbA1C, but not duration of diabetes (14).

Even though a correlation between KL and DN has been reported, the regulation of KL levels in DN is still unclear. miRNAs could be potential candidates as they have been shown to play a critical role in DN. Expression of miRNA-192 is increased in glomeruli isolated from both type 1 and type 2 diabetes animal models compared to their nondiabetic controls (15). Importantly, inhibition of miR-192 or miR-192 knockout results in decreased proteinuria and renal fibrosis in streptozotocin-induced diabetic mouse models (16–18). Urinary miR-192 has been correlated with albuminuria in a cohort of patients with T1D (19).

We hypothesized that KL levels will be affected by diabetes duration and glycemic control in children with Type 1 diabetes, and KL levels could be regulated by miRNAs, specifically miR-192. We studied the relationship of circulating KL levels to patient and metabolic characteristics in children with T1D, and the potential role of miR-192 in regulating the levels of KL.

## Research design and methods

Seventy-nine consecutive pediatric patients (age:10.9-23.9 years) with T1D (duration: 2.1-17.6 years) who met International Society of Pediatric and Adolescent Diabetes (ISPAD) screening criteria for nephropathy screening (≥ 10 years of age and ≥ 2 years of duration) were recruited (20). Serum sample was obtained along with random urine collection at recruitment for measurement of albumin/creatinine ratio (ACR). Demographic, anthropometric, and laboratory data was obtained from the institution’s EMR. The study was approved by the Institutional review board at University of Pittsburgh (Protocol Number: PRO15100286).

### Serum KL level

Serum α-KL level was determined using ELISA kit from IBL (Cat# 27998, Immuno-Biological Labs, Japan) according to the manufacturer’s instructions. The coefficient of variability (%CV) is 2.7-6.5, and the lower detection limit was 6.15 pg/mL.

### Serum miRNA expression

Total RNA was extracted from human serum samples using miRNeasy Serum/Plasma Kit (Qiagen, Germantown, MD), and cDNA was synthesized using miScript II RT kit (Qiagen, Germantown, MD) according to the manufacturer’s instruction. The QuantiTect SYBR Green PCR master mix and miScript Primer assays for miR-192, miR-126, miR21, miR-29a and miR-210 (Qiagen, Germantown, MD) were used to perform miRNA real time quantitative PCR. Relative mRNA levels were calculated by 2−ΔΔCt and normalized to miR-210.

### Cell culture and transfections

Human kidney cell line (HK2) was purchased from ATCC and maintained in Dulbecco’s Modified Eagle Medium/Nutrient Mixture F-12 (DMEM/F-12) supplied with 10% FBS and 1X Penicillin-Streptomycin. Medium was changed every other day. For miRNA transfection, reverse transfection was used. Briefly, miR-192 mimic (Life Technologies) or negative control miRNA (Life Technologies) were diluted in 50uL of opti-MEM in a final concentration of 100nM and 1.25ul of Lipofectamine RNAiMax was added and mixed in 24-well plate. After 20 min,1 X 10^5^ cells were seeded into the plated. Cells were harvested 72-h later for further analysis. For plasmid transfection, HK-2 cells were plated in 24-well plate for overnight. Plasmids were transfected into the cells using Lipofectamine 2000 (Invitrogen) according to the manufacturer’s instructions.

### Dual reporter assay

The full length KL3’UTR in luciferase reporter construct (kl-wt-luc) was a gift from Dr. Gwen King (Creighton University). The miR-192 binding site deletion construct (kl-mut-luc) was cloned using Q5^®^ Site-Directed Mutagenesis Kit Protocol kit (New England Biolabs, Ipswich, MA). The kl-wt-luc or kl-mut-luc construct was co-transfected into HK2 cells with renilla luciferase control (Rluc). The cells were then transfected with either control miRNA or miRNA-192 mimic, or miRNA-192 mimic + inhibitor for 48 hours. The miR-192 inhibitor is a pre-synthesized anti-miR™ miRNA Inhibitor (ThermoFisher. Ambion). Anti-miR™ miRNA Inhibitors are chemically modified, single-stranded nucleic acids designed to specifically bind to and inhibit endogenous miRNA molecules. The cells were harvested in lysis buffer and the luciferase activity was measured using Dual-Luciferase^®^ Reporter Assay kit (Promega, Madison, WI).

### Oxidative stress assay

Oxidative stress was assessed in human serum or cell lysate using Thiobarbituric Acid Reactive Substances kit (Cat# 10009055, Cayman Chemical, Ann Arbor, Michigan) according to the manufacturer’s instruction.

### RNA extraction and real time PCR

Total RNA was extracted from the cells using RNeasy purification kit (Qiagen, Valencia, CA, USA) following the manufacturer’s instructions. First strand cDNA was synthesized from 1 μg total RNA using iScript cDNA Synthesis Kit (Bio-Rad, Hercules, CA, USA). Real-time PCR was carried out using TaqMan assays in a 20 μl reaction mixture (Bio-Rad) containing 0.1 μl first strand cDNA and 1X probe and primers mix (Bio-Rad). Probes were purchased from Life Technologies Relative mRNA levels were calculated by 2−ΔΔCt and normalized to β-actin.

### Western blot

Cells were homogenized in RIPA buffer (50 mmol/l Tris [pH 7.4], 150 mmol/l NaCl, 1% Triton X-100, 0.5% SDS) containing proteinase inhibitors (Roche, Indianapolis, IN, USA) and phosphatase inhibitors. Denatured total protein (30 μg) was resolved on SDS-PAGE, then transferred to PVDF membranes. The membranes were blocked with 5% non-fat dry milk in Tris-buffered saline (154 mmol/l NaCl) with Tween 20 (TBS-T) for 1 h at room temperature. The membranes were incubated with primary antibodies (GAPDH (control) and KL; diluted in 5% BSA in TBS-T) overnight at 4°C. The membranes were then washed three times with TBS-T and incubated with horse-radish peroxidase (HRP)-conjugated secondary antibodies (diluted in 5% non-fat dry milk in TBS-T) for an additional 1 h at room temperature. Images were taken after adding SuperSignal West Dura extended duration substrate (Thermo Fisher Scientific) to the membrane. Antibodies were pre-validated by molecular mass using positive control samples.

### Statistical analysis

Descriptive statistics are presented as frequencies with percentages, means with standard deviation, or medians with interquartile range. Kruskal-Wallis and Wilcoxon Rank Sum tests assessed between group difference for continuous variables. Correlations between KL and other variables was assessed using Spearman rank order correlation analysis. Partial correlations were also computed adjusting for age. Additionally, Chi-square tests, t-tests, were used to assess difference between groups for categorical and continuous variables, respectively. Linear or logistic regression models were used to adjust for age, sex, and relevant covariates. miRNA levels were not normally distributed and thus were log10-transformed for analysis. The relationship between miR-192 and diabetes duration was assessed using locally weighted scatterplot smoothing (LOWESS) regression. Because there was a pronounced inflection point at ~12 years of disease duration, subsequent analyses were stratified by duration <12 vs >12 years. All statistical tests were two-tailed with *P* values of ≤0.05 considered to be statistically significant.

## Results

A total of 79 subjects with type 1 diabetes meeting ISPAD criteria for nephropathy screening were consecutively recruited. Background characteristics are summarized in **Table 1**. Most subjects were white (90%), half were male (51%), mean ± SD age of 16.1 ± 3.1 (range: 10.2-23.9), age at onset of 8.9 ± 3.8 (range: 1.1-17.2), and duration of T1D of 7.2 ± 3.9 (range: 2-18) years. Body mass index (BMI) percentile (median [IQR]) was 73.5 [45.7-88] and BMI standard deviation score (SDS) (median [IQR]): 0.64 [-0.01-1.17]. In terms of laboratory parameters, HbA1c at the time of the evaluation was 8.3 ± 1.5% (range: 7.3-9), 2-year average HbA1c 8.0 ± 1.3% (range: 7.3-8.5), mean eGFR 99.1 ± 19.9 ml/kg/1.73m^2^, median [IQR] albumin/creatinine ratio was 9.8 [5.5, 21.1] and serum soluble α-KL 1204 [871, 1537] pg/ml. There were 13 subjects that had abnormal ACR at the study visit (ACR ≥ 30 mg/gr). Of these, 5 had a prior ACR checked with 3 having an abnormal result (5, 11 and 18 months prior to the study visit). One had been prescribed lisinopril with very poor compliance documented in the medical record. All 13 subjects were included in the analysis.

**Table 1:** Characteristics of the patient cohort.

Variables	
Age (years)	16.1 ± 3.1
Age at onset (years)	8.9 ± 3.8
Diabetes duration (years)	7.2 ± 3.9
Female/Male (%)	49/51
Race (White/Black/Hispanic) (%)	90/9/1
BMI percentile	73.5 [45.7-88]
BMI SDS	0.64 [-0.01-1.17]
HbA1c at time of evaluation (%)	8.3 ± 1.5
HbA1c average of 2 years including time of evaluation (%)	8.0 ± 1.3
Albumin/Creatinine (ACR) (mg/g)	9.8 [5.5, 21.1]
eGFR (ml/kg/1.73 m^2^)	99.1 ± 19.9
Serum soluble α-KL (pg/ml)	1204 [871, 1537]

Data are presented as mean ± SD except for BMI percentile and BMI SDS, ACR and KL (median [25^th^, 75^th^]).

BMI, Body mass index.

SDS, Standard deviation score.

### Serum α-KL is associated with glycemic control and diabetes duration in a pediatric population with T1D

**Figure 1:**
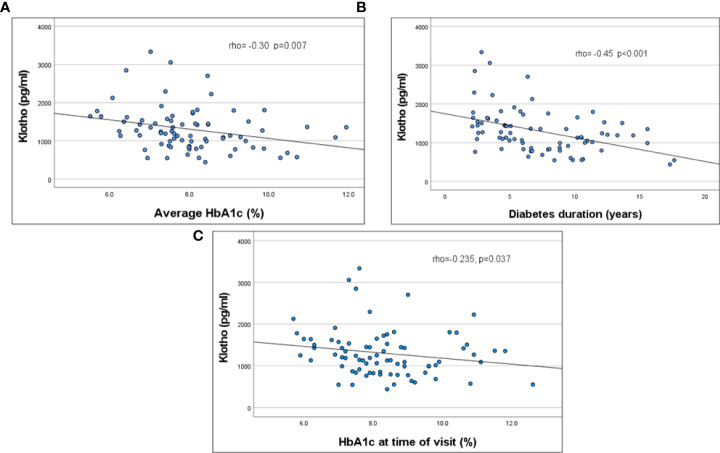
Correlation of serum KL levels to HbA1C (2 years average, **(A)** p =0.007), HbA1C at the time of evaluation (**B**, p=0.037) and duration of diabetes (**C**, p<0.001).

KL was significantly correlated with HbA1c at time of evaluation, average HbA1c over 2 years, and diabetes duration (**Figure 1**; **Table 2**). This remained significant even when adjusted for age. KL did not correlate with ACR. There was no difference in KL levels by quartiles of ACR or between those with normal (ACR < 30 mg/gr) (n=62) vs abnormal (ACR ≥ 30 mg/gr) (n=13) ACR (1165 [868-1503]vs 1355 [912-1715] pg/ml, p= 0.5). There was no significant correlation between KL and age, eGFR, SBP %ile and DBP %ile (**Table 2**).

**Table 2:** Correlations between KL and other independent variables.

Variables	R	p-value	N
Age (years)	0.03	0.79	79
Diabetes Duration (years)	-0.453	**<0.001**	79
A1C at the time of evaluation	-0.235	**0.037**	79
Average HbA1c (last 2 years)	-0.301	**0.007**	79
Average SBP (%ile)	0.005	0.97	72
Average DBP (%ile)	-0.077	0.52	72
BMI (%ile)	-0.59	0.62	72
ACR (mg/g)	0.016	0.89	75
Triglycerides (mg/dl)	-0.116	0.31	79
Total Cholesterol (mg/dl)	-0.108	0.34	79
HLD- Cholesterol (mg/dl)	-0.100	0.38	79
LDL-Cholesterol (mg/dl)	-0.061	0.59	79

Bold indicates a significant difference.

### Serum miRNA 192 levels negatively correlate with serum KL in children with longer duration of diabetes

**Figure 2:**
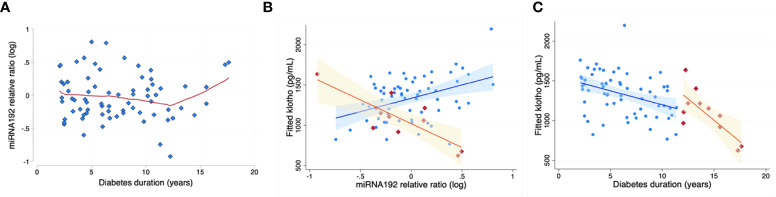
miR-192 levels and KL levels with diabetes duration. miR-192 levels are relatively stable with disease <12 years duration. There is an “inflection point” at ~12 years, with miR-192 levels increasing after that with increased duration of diabetes **(A)**. Red line is based on LOWESS (locally weighted scatterplot smoothing) regression. Correspondingly, in a predictive model using miR-192 level and adjusted for age and sex **(B)**, KL levels decrease more steeply after 12 years of duration of diabetes (orange segment; shaded area represents 95% confidence bands). Blue segment demonstrates the relationship in children with diabetes duration of less than 12 years. **(C)** shows significant inverse relationship between miRNA-192 and KL levels with prolonged duration of diabetes (>12 years of diabetes; orange, r^2 =^ 0.89, p=0.03).

In the study population above, serum expression level of multiple renal function related miRNAs, including miR-192, miR-126, miR-21, miR-29a and miR-210 were estimated (15, 17, 21–23). Circulating miR-192 levels tended to increase with age (p=0.051), but did not correlate with HbA1C, duration of diabetes, BMI percentile, BMI z score, systolic BP, diastolic BP, Total cholesterol, HDL, LDL or VLDL levels. miR -192 levels were relatively stable among subjects of shorter diabetes duration, with a clear inflection point at 12 years of diabetes, after which miR-192 levels increased with time (**Figure 2A**). Moreover, miR-192 levels negatively correlated with KL levels in children with prolonged duration of diabetes (>12 years) (**Figure 2B**). After adjustment for age and sex, KL decreased -246.8 pg/ml (95% CI -412.0 to -81.6) per each 1-unit increase in miR-192 relative ratio (n=11, p=0.01, model R2 = 0.65) (**Figure 2C**). There was no relationship between miR-192 and HbA1C (data not shown). Levels of miR-126, miR21, miR-29a and miR-210 did not correlate with HbA1C, duration of diabetes, BMI percentile. BMI z score, systolic BP, diastolic BP, Total cholesterol, HDL, LDL, VLDL levels or KL levels in our population.

### Overexpression of miR-192 in HK2 cells inhibits the expression of KL

**Figure 3:**
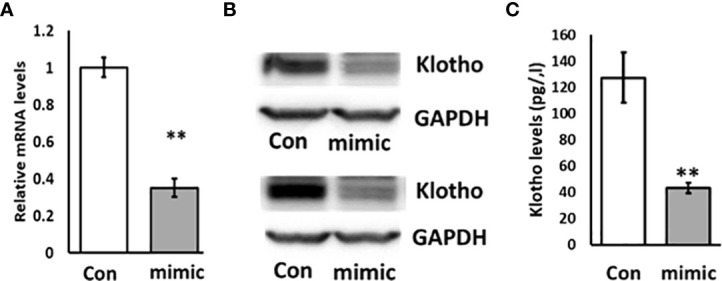
Overexpression of miR-192 inhibits the expression of KL in HK2 cells. miR-192 mimic or scrambled miRNA were transfected into HK2 cells for 72 hours and mRNA **(A)**, protein **(B)** and soluble KL in the media **(C)** were detected by real time PCR, immunoblotting and ELISA. **p < 0.01.

To investigate whether miR-192 regulates the expression of KL, we transfected miR-192 mimic into HK2 cells for 72 hours. We found that the expression level of KL is significantly suppressed in HK2 cells by miR-192 mimic transfection (**Figure 3A**). We also found that the protein level of KL is markedly inhibited by miR-192 mimic compared to control (scrambled miRNA) transfection (**Figure 3B**). In addition, the soluble KL levels in the media, measured using ELISA assay, is significantly decreased in cells transfected with miR-192 mimic (**Figure 3C**). This demonstrated that miR-192 regulates KL expression at both mRNA and protein levels.

### miR-192 inhibitor mitigates the effects of overexpression of miR-192 in HK2 cells on oxidative stress, inflammation, and senescence

**Figure 4:**
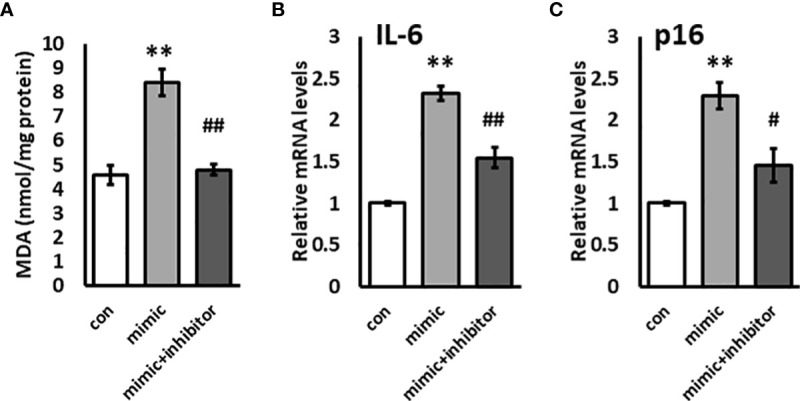
Overexpression of miR-192 induces oxidative stress and inflammation in HK2 cells. miR-192 mimic, mimic + inhibitor, or scrambled miRNA were transfected into HK2 cells for 72 hours and MDA levels in the cell lysate **(A)**, and mRNA levels of IL-6 **(B)** and p16 **(C)** were measured. **p<0.01, #p<0.05, ##p<0.01.

KL plays a critical role in oxidative stress, inflammation, and senescence. Since miR-192 mimic transfection resulted in a dramatic reduction in KL levels, we examined oxidative stress using the TBARS assay for MDA in HK2 cells transfected with miR-192 mimic. We found that transfection of miR-192 mimics causes a 2-fold increase in oxidative stress in HK2 cells (**Figure 4A**). We also examined the gene expression levels of IL-6 and p16, markers of inflammation and a senescence, and we found that IL-6 and p16 are both significantly upregulated in cells transfected with miR-192 mimics (**Figure 4B, C**). The co-transfection of miR-192 mimic and the inhibitor mitigated the effects of miR-192 on oxidative stress, inflammation, and senescence, and significantly decreased the levels of MDA, IL-6 and p16 levels (**Figures 4A–C**). These experiments demonstrate that miR-192 affects oxidative stress, inflammation, and senescence in HK2 cells.

### KL is a potential target of miR-192

**Figure 5:**
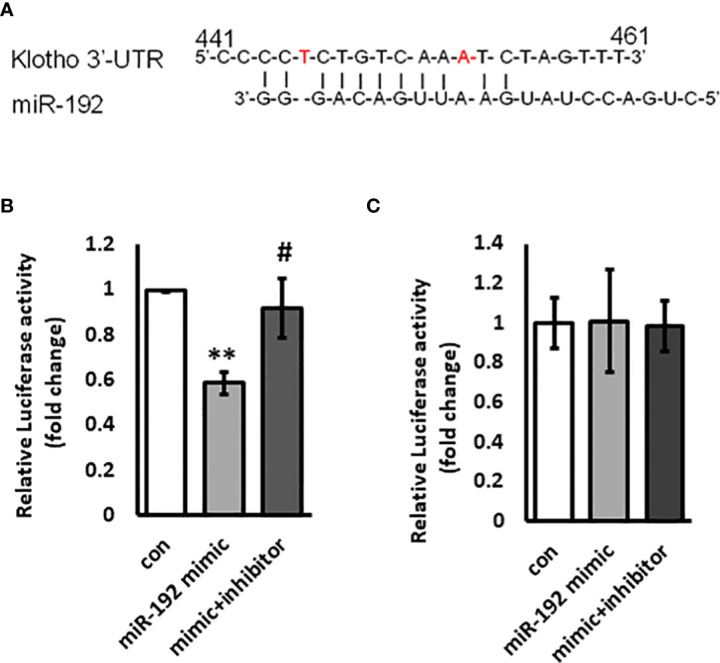
KL is a target gene of miR-192. Potential miR-192 binding site on the 3’UTR of KL promoter is shown in **(A)**. miR-192 mimic or scrambled miRNA or the combination of miR-192 mimic and miR-192 inhibitor were co-transfected with kl-wt-luc 3’UTR reporter **(B)** or kl-mut-luc 3’UTR reporter **(C)** into HK2 cells for 72 hours and reporter activity was measured using Dual-Luciferase Reporter Assay kit. **p<0.01 and #p<0.05.

miRNAs largely regulate gene expression through directly binding to the 3’UTR of target genes and inducing degradation of mRNAs or decreased mRNA translation. Since miR-192 overexpression suppresses the mRNA levels of KL, we speculate that KL is a miR-192 target gene in kidney cells. We scanned the 3’UTR sequence of KL mRNA and found a potential binding site for miR-192 in the 3’UTR of KL mRNA (**Figure 5A**). To further examine whether miRNA-192 directly regulates KL expression, we co-transfected miR-192 mimic or the combination of mimic and inhibitor together with the kl-wt-luc or kl-mut-luc reporter constructs. We then performed the dual luciferase reporter assay and found that miR-192 mimic inhibits wt KL 3’UTR luciferase activity, and that the inhibitor abolishes the effects of the mimic (**Figure 5B**). Furthermore, both mimic and inhibitor lost their effects on the kl-mut-luc 3’UTR reporter construct (**Figure 5C**), suggesting that miRNA-192 regulates KL expression specifically through binding to the novel miR-192 binding site in the KL 3’UTR.

## Discussion

This is the first study to date to examine levels of KL, miR-192 and their relationship with established biochemical and clinical markers of diabetes and its complications in youth with type 1 diabetes. Our results show that circulating KL levels are negatively correlated to HbA1c and diabetes duration after adjusting for age. Serum levels of miR-192 are negatively associated with circulating KL levels in children with prolonged duration of diabetes, suggesting a regulatory role of miR-192 in the expression of soluble KL. This was confirmed by *in vitro* experiments that demonstrated the role of miR-192 in regulating KL expression and levels. We show that miR-192 is an upstream regulator of KL, and induces markers of oxidative stress, inflammation, and senescence *in vitro*. Finally, we demonstrate that KL is a direct target gene of miR-192.

Despite the wealth of information on biomarkers of diabetes complications in adults with T1D, data in the pediatric population with T1D are limited. DN, one of the most serious chronic complications of T1D and the leading cause of mortality (24), could be potentially missed in children, as albuminuria (the current non-invasive “gold” standard for screening, monitoring, and predicting progression of DN in adolescents) may be temporary and may not necessarily reflect permanent renal impairment (25). It is thought that renal hyperfiltration may be the earliest hemodynamic abnormality seen in patients with diabetes and it has been linked with an increased risk of DN with rapid decrease in glomerular filtration rate (GFR) (26). Moreover, several lines of evidence suggest that advanced lesions in both glomerular and tubular structures may be present in non-albuminuric subjects with T1D (27). These observations have raised significant concern in the use of albuminuria alone as a reliable marker for risk of development of DN. Thus, a major pitfall has been the inability to identify high-risk patients at an early stage for microvascular and other complications. Interestingly, studies have shown that there is no significant difference in serum KL in patients with diabetes without nephropathy compared to healthy controls, whereas soluble KL is decreased in early chronic kidney disease in diabetic patients (28). Our study shows that KL is negatively correlated significantly with HbA1c, and this is consistent with a previous study from Poland in a pediatric population. Contrary to that same study, we found a significant negative correlation with diabetes duration, likely because our population had a longer duration of T1D (14). Poor glycemic control and prolonged duration of diabetes are the most important risk factors for development of complications, and a decrease in KL levels may reflect early changes at the renal level which may not be identified by albuminuria. This is supported by a recent meta-analysis showing that circulating KL levels were lower even in the very early stages of DN (29). Based on these, circulating levels of KL could be used as an early marker for DN in youth with T1D. Furthermore, overexpression of the KL gene or administration of KL offers beneficial effects in rodent models of various renal diseases (30), including db/db mice (31), suggesting that induction of KL could be a novel therapeutic strategy for treating DN.

Many recent studies have shown that miRNAs play critical roles in DN. Thus, in addition to the circulating levels of KL, we examined the serum levels of multiple miRNAs that had previously been implicated in DN, including miR-192, miR-126, miR-21, miR-29a and miR-210. Of these, only miR-192 is negatively correlated with circulating levels of KL in children with prolonged duration of diabetes, which we expect is associated with an increased risk for diabetic complications. This is broadly consistent with prior observations that levels of miR-192 are negatively correlated with albuminuria in patients with type 2 diabetes (32). Elevations of TGF-β and miR-192 have been shown to lead to increased Col1a2 expression (15, 17) and attenuation of autophagy in glomerular mesangial cells (MMC) *in vitro* (16). It has been shown that TGF-β induces renal fibrosis by increasing miR-192 expression, and miR-192 inhibition blocks TGF-β induced renal fibrosis in a mouse model of chronic kidney disease (33). On the other hand, reduced levels of KL aggravates renal fibrosis in chronic kidney disease (34), and overexpression of KL reduces senescence and oxidative stress, and decreases fibrosis and kidney injury in mice in a model of immune complex glomerulonephritis (11). Our results show a role for miR-192 in oxidative stress, inflammation and senescence, key pathogenic mechanisms in the development of DN. Interestingly, even though both miR-192 and KL have been independently implicated in the pathogenesis of DN, the relationship between miR-192 and KL has not yet been reported. In this study, we show that overexpression of miR-192 inhibits the levels of KL in HK2 cells, and that miR-192 targets KL through its 3’UTR suggesting that KL is a target gene of miR-192.

In summary, our studies provide evidence that in children with T1D, changes in miR-192 and KL correlate with risk factors for complications such as DN, and that changes in KL and/or miR-192 levels could serve as early biomarkers for diabetes complications in youth with T1D. Our *in vitro* experiments provide proof of concept for a role of miR-192 in influencing KL levels, as well as pathophysiological processes such as oxidative stress, inflammation or senescence that play a role in diabetes complications. Limitations of our study include a lack of normal control subjects, and a smaller sample size of subjects with prolonged duration of diabetes. Future longitudinal follow up studies are needed to establish that lower KL levels preceded the onset of DN in our cohort of children with T1D.

## Data availability statement

The original contributions presented in the study are included in the article/supplementary material. Further inquiries can be directed to the corresponding author.

## Ethics statement

The studies involving human participants were reviewed and approved by the Institutional review board at University of Pittsburgh (Protocol Number: PRO15100286). Written informed consent to participate in this study was provided by the participants’ legal guardian/next of kin.

## Author contributions

ZG, IL, JH, RM-study design. ZG, YL, PP- performed the experiments. ZG, IL, PP, VA, HF, EF, JH, RM- analysis of data and interpretation. ZG, IL, EF, JH and RM- writing the manuscript. All authors contributed to the article and approved the submitted version.

## Funding

This project was supported in part by the Children’s Hospital of Pittsburgh (RM), Cochrane-Weber Endowed Fund grant (ZG, RM, PP) and NIH T32 grant DK007729 (PP).

## Acknowledgments

The authors thank Dr. Gwen King (Creighton University) for providing the full-length klotho 3’UTR reporter construct.

## Conflict of interest

The authors declare that the research was conducted in the absence of any commercial or financial relationships that could be construed as a potential conflict of interest.

## Publisher’s note

All claims expressed in this article are solely those of the authors and do not necessarily represent those of their affiliated organizations, or those of the publisher, the editors and the reviewers. Any product that may be evaluated in this article, or claim that may be made by its manufacturer, is not guaranteed or endorsed by the publisher.
